# Long-term trends in *Anopheles gambiae* insecticide resistance in Côte d’Ivoire

**DOI:** 10.1186/s13071-014-0500-z

**Published:** 2014-11-28

**Authors:** Constant AV Edi, Benjamin G Koudou, Louise Bellai, Akre M Adja, Mouhamadou Chouaibou, Bassirou Bonfoh, Sarah JE Barry, Paul CD Johnson, Pie Müller, Stefan Dongus, Eliezer K N’Goran, Hilary Ranson, David Weetman

**Affiliations:** Liverpool School of Tropical Medicine, Pembroke Place, Liverpool, L3 5QA UK; Centre Suisse de Recherches Scientifiques en Côte d’Ivoire, 01 BP 1303 Abidjan 01, Côte d’Ivoire; Université Nangui-Abrogoua, UFR Sciences de la Nature, 02 BP 801 Abidjan 02, Côte d’Ivoire; Epidemiology and Public Health Department, Swiss Tropical and Public Health Institute, Socinstrasse 57, PO Box, CH-4002, Basel, Switzerland; University of Basel, Petersplatz 1, CH-4003 Basel, Switzerland; Robertson Centre for Biostatistics, University of Glasgow, Boyd Orr Building, Glasgow, G12 8QQ UK; Boyd Orr Centre for Population and Ecosystem Health, Institute of Biodiversity, Animal Health and Comparative Medicine, University of Glasgow, Graham Kerr Building, Glasgow, G12 8QQ UK; Université Felix Houphouët-Boigny de Cocody, Abidjan, 22 BP 582 Côte d’Ivoire; Institut Pierre Richet (IPR), Abidjan, BP 47 Côte d’Ivoire

**Keywords:** Malaria intervention, Pyrethroid, Carbamate, Organochlorine, Organophosphate, Multiple resistance, *kdr*

## Abstract

**Background:**

Malaria control is heavily dependent on the use of insecticides that target adult mosquito vectors via insecticide treated nets (ITNs) or indoor residual spraying (IRS). Four classes of insecticide are approved for IRS but only pyrethroids are available for ITNs. The rapid rise in insecticide resistance in African malaria vectors has raised alarms about the sustainability of existing malaria control activities. This problem might be particularly acute in Côte d’Ivoire where resistance to all four insecticide classes has recently been recorded. Here we investigate temporal trends in insecticide resistance across the ecological zones of Côte d’Ivoire to determine whether apparent pan-African patterns of increasing resistance are detectable and consistent across insecticides and areas.

**Methods:**

We combined data on insecticide resistance from a literature review, and bioassays conducted on field-caught *Anopheles gambiae* mosquitoes for the four WHO-approved insecticide classes for ITN/IRS. The data were then mapped using Geographical Information Systems (GIS) and the IR mapper tool to provide spatial and temporal distribution data on insecticide resistance in *An. gambiae sensu lato* from Côte d’Ivoire between 1993 and 2014.

**Results:**

Bioassay mortality decreased over time for all insecticide classes, though with significant spatiotemporal variation, such that stronger declines were observed in the southern ecological zone for DDT and pyrethroids than in the central zone, but with an apparently opposite effect for the carbamate and organophosphate. Variation in relative abundance of the molecular forms, coupled with dramatic increase in *kdr* 1014F frequency in M forms (*An. coluzzii*) seems likely to be a contributory factor to these patterns. Although records of resistance across insecticide classes have become more common, the number of classes tested in studies has also increased, precluding a conclusion that multiple resistance has also increased.

**Conclusion:**

Our analyses attempted synthesis of 22 years of bioassay data from Côte d’Ivoire, and despite a number of caveats and potentially confounding variables, suggest significant but spatially-variable temporal trends in insecticide resistance. In the light of such spatio-temporal dynamics, regular, systematic and spatially-expanded monitoring is warranted to provide accurate information on insecticide resistance for control programme management.

**Electronic supplementary material:**

The online version of this article (doi:10.1186/s13071-014-0500-z) contains supplementary material, which is available to authorized users.

## Background

Preventing malaria transmission by targeting the main vectors remains a challenge for national malaria control programmes [1]. Only four classes of insecticides, pyrethroids, organochlorines (DDT), carbamates and organophosphates are currently approved for indoor residual spraying (IRS) and only pyrethroids for insecticide treated bednets (ITNs) and long-lasting insecticidal nets (LLINs). High coverage with IRS and especially ITNs has contributed to the decrease in the number of malaria cases throughout sub-Saharan Africa over the past decade [2,3]. However, resistance to pyrethroids and DDT is now widespread in many African countries [4]. Though currently less common, resistance to carbamates and organophosphates, the available alternatives for IRS, is also being reported [5-8], and resistance to all four classes has now been found in wild mosquitoes from southern Côte d’Ivoire [7]. In recognition of this alarming situation, the WHO recently launched the Global Plan for Insecticide Resistance Management [3] with five strategic pillars, the first two of which are (i) planning and application of insecticide resistance management strategies; and (ii) surveillance of resistance. Both of these pillars require data on the presence and dynamics of insecticide resistance at country level.

In Côte d’Ivoire, malaria incidence is monitored routinely at six sentinel sites distributed across the country; insecticide resistance, however, is not monitored regularly. Resistance was monitored only one time in Man [9] and Abengourou [9], respectively; two times in Yamoussoukro [9,10] and San Pedro [9,11] and four times in Abidjan [9,10,12,13] and five times in Korhogo [9,10,12-14].

The strategy of the National Malaria Control Programme towards decreasing and eliminating malaria following the recent civil war period is to integrate approaches targeting both malaria parasites and vectors. This strategy includes malaria diagnostic testing and treatment with artemisinin combination therapy (ACT), increased coverage of and accessibility to LLINs, treatment of larval breeding sites, and more regular monitoring and surveillance of insecticide resistance. In Côte d’Ivoire, where IRS is not currently implemented, ITNs and more recently LLINs remain the main control measure. In 2012, estimated coverage of LLINs had risen to approximately 60% from near-zero just six years earlier [2].

Côte d’Ivoire has a relatively long history of insecticide resistance studies. Indeed, the first cases of pyrethroid and carbamate resistance in wild malaria vectors were reported from Central Côte d’Ivoire in the early 1990s and early 2000s, respectively [15,16]. Recent studies have reported resistance to other insecticide classes across the country [13,17] and even to all WHO-approved classes [7]. Such observations can give the perception that resistance is increasing ubiquitously across insecticides and regions, and effects of temporal variation in research effort and reporting biases are rarely considered. In this study we aimed to use relatively extensive historical published and unpublished literature, and recent field tests on insecticide resistance in *Anopheles gambiae s.l.* mosquitoes, to investigate whether over the last 20 years in Côte d’Ivoire: (1) insecticide resistance has increased; (2) whether any trends are consistent across insecticides and ecological zones; (3) multiple insecticide resistance (across insecticide classes) has increased.

## Methods

### Study sites

**Figure 1:**
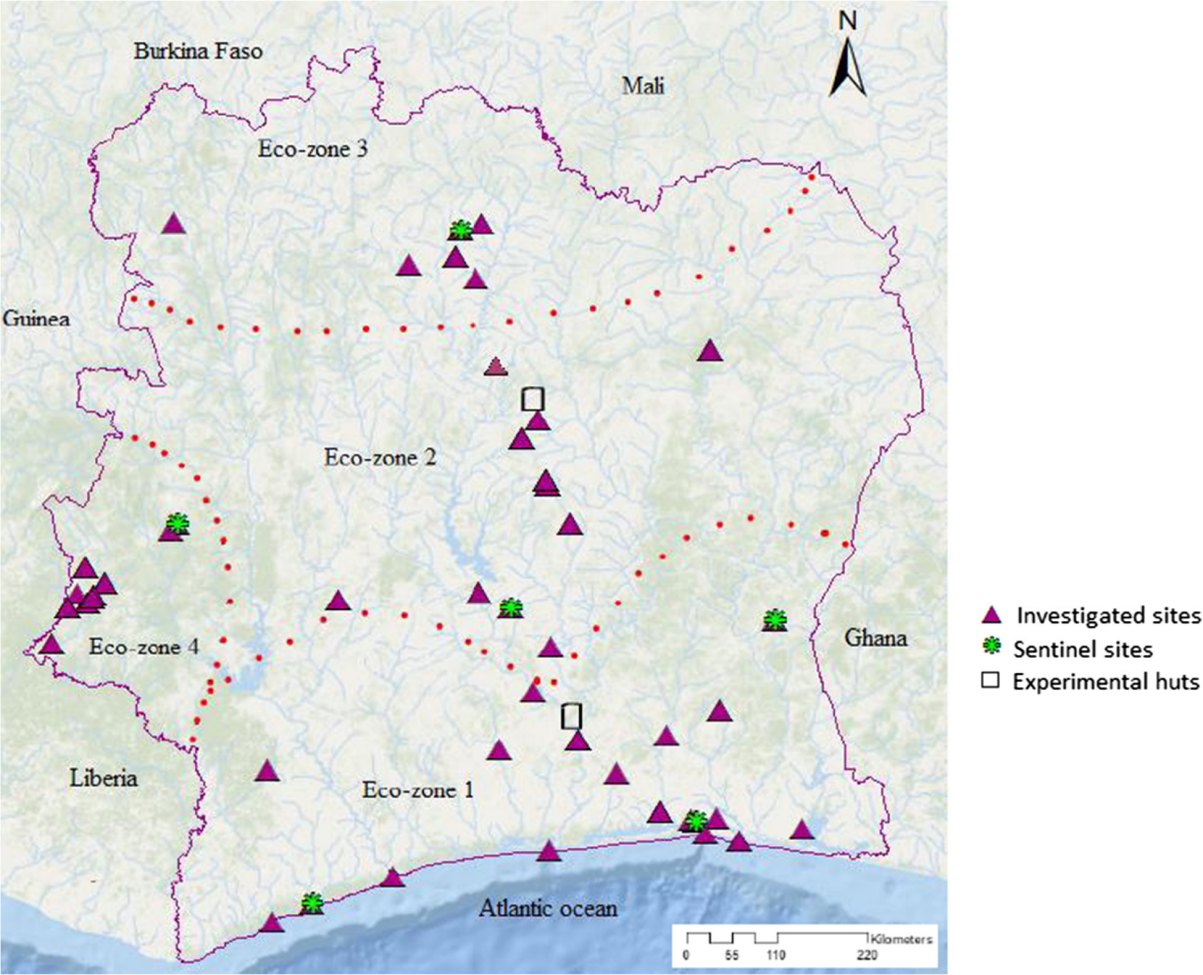
**Map of Côte d’Ivoire showing the distribution of main ecological zones.** Modified from “Ecoregions of Côte d’Ivoire”. Source: World Wildlife Fund. Encyclopaedia of earth: http://www.eoearth.org/view/article/151626/.

Côte d’Ivoire is a West African country of 322,462 square kilometres and 22 million inhabitants. It is bordered by Burkina Faso and Mali in the North, Liberia and Guinea in the West, Ghana in the East and the Atlantic Ocean in the South. Seasons are distinguishable by rainfall and temperature. The average temperature increases from 25°C in the South to 30°C in the North [18]. The average humidity increases from 71% in the North to 85% in the South. The climate is equatorial in the southern coasts and tropical in the centre to semi-arid in the far north and there are three seasons: warm and dry (November to March), hot and dry (March to May), hot and wet (June to October). The country is divided into four ecological zones (Figure 1) based on climate data [19]. The first ecological zone (involving all of the southern region) is characterized by equatorial transition climate (Guinean or Attiéan climate) with annual rainfall between 1,300 and 2,400 mm. Dense moist forest is the characteristic vegetation found in this zone. In ecological zone 2 (the centre, and central north), there is an attenuated equatorial transition climate (Baoulean climate). The annual rainfall ranges between 1,500 and 2,200 mm. Vegetation is characterized by Guinean forest-savannah mosaic belt (forest and southern part of the savannah). The third ecological zone (the North) belongs to tropical transition climate (Sudanian climate) with annual rainfall between 1,100 and 1,700 mm. Vegetation is represented by savannah. In the fourth ecological zone (the West), there is a mountain climate (A subequatorial climate) with two seasons and annual rainfall between 1,500 and 2,200 mm. Vegetation is characterized by evergreen forest.

### Literature review

A systematic search of all published and non-published papers on insecticide resistance in Côte d’Ivoire was carried out. All studies carried out within the period covering 1993 to 2014 in which insecticide resistance was monitored using WHO tube assays in *An. gambiae s.l.* [20] were selected for analysis. We used various sources including IR mapper, PubMed, MSc and PhD theses from libraries of research institutes and national universities. Data were obtained from 52 published materials, 1 MSc thesis, and unpublished data from the AvecNet programme (www.avecnet.eu) collected between May 2013 and May 2014 (Additional file 1). The following variables were recorded from each source: collection sites; latitude and longitude; collection date; insecticides tested; *An. gambiae* M and S molecular forms (*An. gambiae s.s.* and *An. coluzzii*, respectively); target–site mutation frequencies; temperature and relative humidity data.

### New bioassay data

Within the AvecNet programme, *An. gambiae* mosquitoes were collected as larvae and reared to adults as described previously [7]. Mosquitoes were exposed to five insecticides representing all four insecticide classes used for ITN and IRS control (the carbamate bendiocarb, the organophosphate fenitrothion, the pyrethroids permethrin and deltamethrin, and the organochlorine DDT) using the standard WHO insecticide susceptibility test [20]. However, for fenitrothion the WHO protocol has very recently been changed to a two hour rather than the one hour exposure we applied [20]. Mortality was recorded 24 hours after insecticide exposure at 25°C and 70–80% relative humidity. For each insecticide it was aimed to have 100 (in batches of 25 mosquitoes per cylinder) females of the age between 2 and 5 days and 50 (in batches of 25 mosquitoes per cylinder) mosquitoes were exposed to non-treated papers as a negative control alongside the test exposures. Data are shown in Additional file 1.

### Data analysis

Geographic coordinates provided in publications were double-checked through the Directory of Cities and Towns in the World (1996–2010) website [21]. Data was imported into ArcGIS (ESRI, Redlands, CA) software version 10.2. Generalized Linear Models with a binary logistic link function were run in SPSS 20 to test the effect of year of sample, ecological zone and their interaction on bioassay mortalities. For permethrin and carbamates we also included insecticide concentration and formulation, respectively, as additional factors (see below). For each model we only included data from ecological zones 1 and 2 because of a paucity of temporal variance in data points in zones 3 and 4. For DDT all studies used the standard WHO diagnostic dose of 4% and for deltamethrin only those studies using the standard concentration of 0.05% were included for analysis. For permethrin, older studies tended to use a 1% concentration, whereas, newer studies used the WHO standard of 0.75%. Therefore, we included bioassay data using each of these two concentrations but when analysing permethrin alone we included concentration as an additional factor in the model. Data were more limited for carbamates and organophosphates, and for the former we included both bendiocarb and propoxur, but excluded carbosulfan owing to much lower mortality, and included insecticide formulation as a factor in the carbamates model. For organophosphates we included both fenitrothion and malathion owing to comparable mortalities, though inclusion of malathion had little impact on results because of very few data points. SPSS 20 was used to calculate Pearson correlation coefficients to illustrate plots of mortality vs. time for each ecological zone; Spearman rank correlations were also calculated but are only reported if they provided a better fit to the data. Statistics for *kdr* data are based on unweighted frequencies from each study site, with t-tests used to compare frequencies.

## Results

### Bioassay data and species distributions

A total of 323 data points were obtained from studies conducting insecticide resistance bioassays using WHO tubes [20], originating from 57 collection sites covering the period from 1993 to 2014 (Additional file 2a). Data were stratified according to the four ecological zones of the country (see Figure 1), with the majority of records from zones 1 (32%) and 2 (49%). Pyrethroid and organochlorine susceptibility was tested in 56 sites (Additional file 3 and Additional file 4); organophosphates and carbamates in 24 sites (Additional file 5 and Additional file 6) almost entirely located in ecological zones 1 and 2. Bioassays were performed using a total of 16 insecticide formulations of which the pyrethroids permethrin and deltamethrin and the organochlorine DDT were most frequently tested (Additional file 2b).

### Interspecific (interform) variation in distribution and insecticide resistance

**Figure 2:**
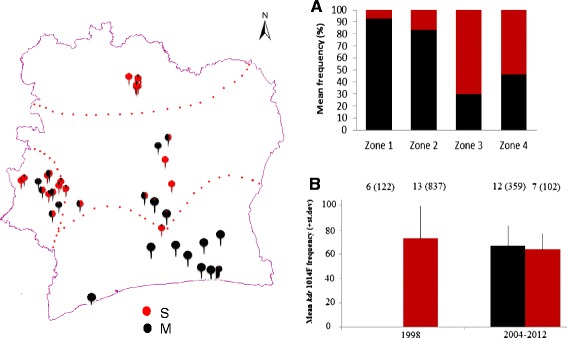
**Distribution of** ***An. gambiae*** **M (black) and S (red) molecular forms across the four ecological zones of Côte d’Ivoire; each recording is shown by a separate point and mean frequency prevalence described in Plot (A).** Mean *kdr* 1014F frequency across sample sites in each form in early samples (from 1998) and more recent samples (2004–2012); and sample sites (and total numbers genotyped) are shown at the top of the plot **(B)**.

The frequency of the M and S molecular forms (i.e. *An. coluzzii* and *An. gambiae s.s.*) varied sharply across the ecological zones of Côte d’Ivoire (Figure 2A) with a predominance of M forms in zone 1 and often high frequencies in zone 2, but a majority of S forms in zones 3 and 4. Relatively few studies recorded insecticide susceptibility data separately for each molecular form, and data were insufficient for any single insecticide to conduct any quantitative analysis of possible differences in phenotypic resistance between the molecular forms (Additional file 2c). However, molecular resistance diagnostic data for the *kdr* L1014F polymorphism (Additional file 2d), originating primarily from two geographically wide-ranging studies [13,22] highlight a temporal discordance between the molecular forms (Figure 2B). For S forms average frequencies recorded in 1998 and 2004–2012 were similarly high (t-test, P = 0.41). For M forms *kdr* 1014F was entirely absent from the six sites surveyed in 1998 (N = 122 genotyped), but in later collections (from 2004–2012) present at an equivalent frequency to both the early and late collections of S forms (t-tests, P > 0.5 for both comparisons). Therefore, any resistance phenotype mediated by *kdr* 1014F would be expected to have increased more sharply in M than S forms.

### Spatio-temporal analysis of resistance across insecticides

**Figure 3:**
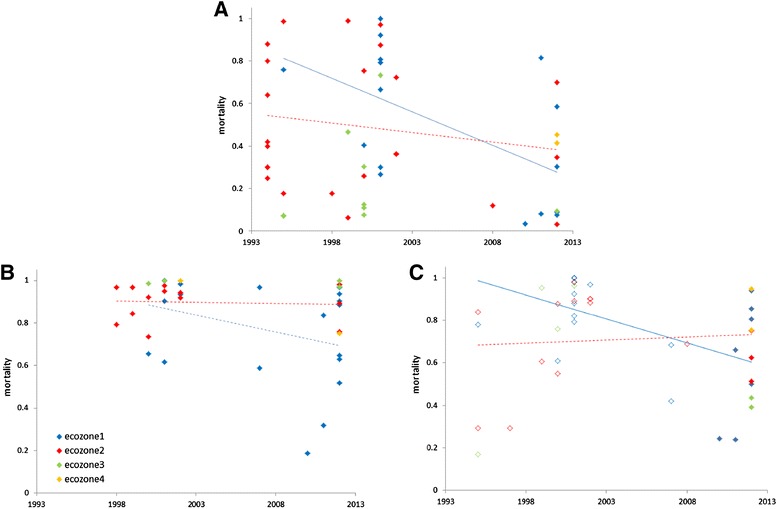
**Temporal trends in mortality to (A) DDT; (B) deltamethrin and (C) permethrin in the four ecological zones of Côte d’Ivoire.** Solid lines indicate significant correlations, dashed lines are non-significant. In all plots quantitative analysis was performed only on ecozones 1 and 2. In all analyses there was a significant effect of year and year × ecozone on mortality. In **(C)** open symbols show data for bioassays with a concentration of 1% and filled circles with the current WHO standard of 0.75%.

**Figure 4:**
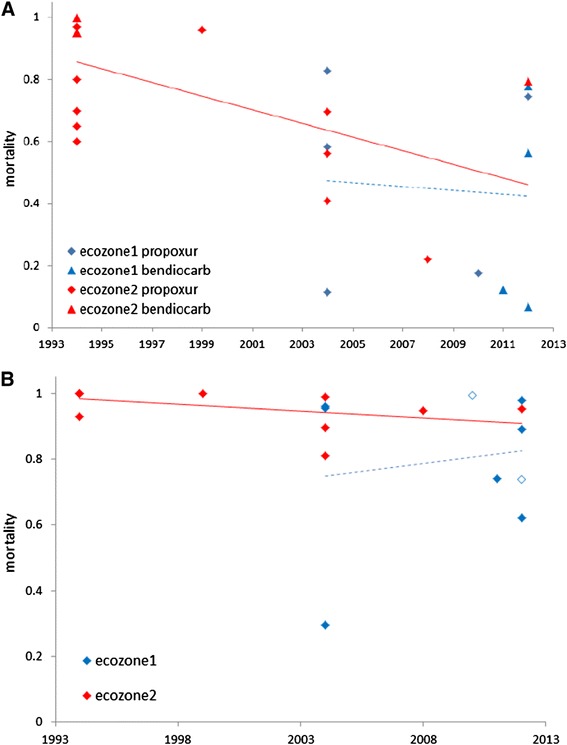
**Temporal trends in mortality to (A) carbamates (propoxur and bendiocarb) and (B) organophosphate (fenitrothion, barring two open symbols for malathion) in the southern and central ecological zones of Côte d’Ivoire.** Solid lines indicate significant correlations, dashed lines are non-significant. In both analyses there was a significant effect of year x ecozone on mortality.

We used a binomial generalized linear model (GLiM) to investigate temporal variation in bioassay mortality across insecticides and ecological zones (Table 1). All main effect and interaction terms in the model were highly significant, suggesting inconsistencies in temporal trends among insecticides and zones. We therefore proceeded by analysing each insecticide separately, including additional terms in the models where necessary (see Methods). Mortality to DDT was very variable across sampling years but with resistance ubiquitous in bioassays conducted after the break (2003–2007) due to the political crisis in the country (Figure 3A). This temporal decline was highly significant as was the interaction between year and ecological zone (Table 2a), evident from a significant negative correlation for zone 1 (r = −0.56, P = 0.025, n = 16) but not zone 2 (r = −0.17, P = 0.42, n = 24). Deltamethrin typically yielded far higher mortality than DDT, with some susceptibility, or at least low-prevalence resistance detected in all ecological zones in recent bioassays (Figure 3B). Again there was a highly significant temporal decline, albeit representing a more modest decrease in resistance, with the same trend in variation between zones; significant as an interaction in the GLiM but not as a correlation across samples (ecozone 1: r = −0.34, P = 0.15, n = 20; ecozone 2: r = −0.11, P = 0.65, n = 20). Similarly for permethrin, there was a significant interaction between year and ecological zone (Table 2c), manifested again as a far more pronounced temporal decrease in mortality in ecological zone 1 (Figure 3C). However, insecticide concentration (0.75% *vs.* 1%) explained the most variance in the model (Table 2c), with higher concentration yielding higher mortality as expected, and a possible interaction with time could not be evaluated because higher concentrations were only used in earlier studies (Figure 3C). Although both bioassay concentrations were applied in each ecological zone, we cannot rule out some confounding effect of concentration on the apparent difference between zones in temporal trends. Analysis for carbamates, for which the difference between propoxur and bendiocarb was significant (Table 2d) but small, and organophosphate bioassay mortality (Table 2e) must be treated with caution owing to more limited data, especially with respect to the lack of early studies in ecological zone 1. Nevertheless, results appear similar for each class (Figure 4A,B, with a significant decline in mortality only in ecological zone 2 for carbamates (ecozone 1: r = −0.08, P = 0.85, n = 9; ecozone 2: r = −0.59, P = 0.014, n = 17) and organophosphates (ecozone 1: r = 0.16, P = 0.73, n = 7; ecozone 2: ρ = −0.63, P = 0.027, n = 12), and therefore effectively opposite to those for DDT and pyrethroids.

**Table 1:** **Generalized linear model testing the effects of sampling year (1993–2012), insecticide (DDT, permethrin, deltamethrin, bendiocarb & propoxur, fenitrothion & malathion), ecological zone (zones 1 and 2) and their interactions on bioassay mortality**

**Model term**	**Wald χ** ^**2**^	**d.f.**	**P-value**
Intercept	157.498	1	0.000
Year (covariate)	154.101	1	0.000
Insecticide	90.866	4	0.000
Ecological zone	30.381	1	0.000
Insecticide × year	92.643	4	0.000
Ecological zone × insecticide	31.005	1	0.000
Insecticide × ecological zone	223.041	4	0.000

A three-way interaction term could not be fitted to model owing to insufficient variance in one of the combinations.

**Table 2:** **Generalized linear model testing the effects of sampling year (1993–2012), ecological zone (zones 1 and 2) and their interaction on bioassay mortality for (a) DDT, (b) permethrin, (c) deltamethrin, (d) carbamates (bendiocarb & propoxur), (e) organophosphates (fenitrothion & malathion)**

**(a) DDT**	**Wald χ** ^**2**^	**d.f.**	**P-value**
Intercept	337.471	1	0.000
Year (covariate)	337.866	1	0.000
Ecological zone	68.178	1	0.000
Year × ecological zone	68.062	1	0.000
**(b) deltamethrin**	**Wald χ** ^**2**^	**d.f.**	**P-value**
Intercept	56.854	1	0.000
Year (covariate)	55.615	1	0.000
Ecological zone	4.833	1	0.028
Year × ecological zone	4.926	1	0.026
**(c) permethrin**	**Wald χ** ^**2**^	**d.f.**	**P-value**
Intercept	6.848	1	0.009
Year (covariate)	7.001	1	0.008
Insecticide concentration	63.232	1	0.000
Ecological zone	33.974	1	0.000
Year × ecological zone	33.889	1	0.000
**(d) carbamate**	**Wald χ** ^**2**^	**d.f.**	**P-value**
Intercept	1.205	1	0.272
Year (covariate)	1.193	1	0.275
Insecticide type	5.922	1	0.015
Ecological zone	72.697	1	0.000
Year × ecological zone	72.311	1	0.000
**(e) organophosphate**	**Wald χ** ^**2**^	**d.f.**	**P-value**
Intercept	1.517	1	0.218
Year (covariate)	1.693	1	0.193
Ecological zone	59.664	1	0.000
Year × ecological zone	59.063	1	0.000

In (b) and (c) two (similar) insecticide concentrations were present in the data so this term was also included as an effect in each model but not in interactions owing to insufficient variance.

### Multiple resistance

**Figure 5:**
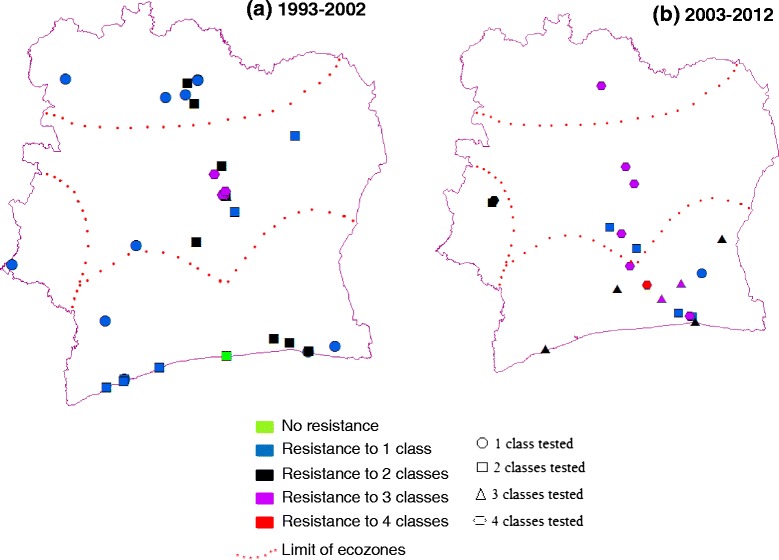
**Records of resistance to different insecticides classes in relation to the number of classes tested in the two decades (a) and (b) spanning 1993 to 2012.** Inset key shows how colours and shapes of the collection site markers illustrate the number of insecticide classes to which the mosquitoes were resistant and the number tested, respectively.

Casual observation of records of resistance to single and multiple insecticide classes (Figure 5) might suggest a temporal increase in the prevalence of multiple resistance, with more records of resistance to two, and especially three or more classes in the second decade (26% vs. 64% of studies in 1993–2002 and 2003–2012, respectively, represented by violet and red colours in Figure 5). However, even without considering geographical variation in sample sites between decades, closer examination reveals differences in bioassay effort (symbol shapes in Figure 5), with a significant shift toward testing of more insecticide classes in more recent studies (χ^2^ = 8.1, 3 d.f., P = 0.04). Consequently, it is not possible to conclude that there has been a significant overall increase in multiple resistance. Moreover, whilst the first record of resistance to all classes emerged in recent years, resistance to three classes was already present in ecological zone 2 prior to 2003.

## Discussion

In this study we aimed to use the relatively long history of WHO bioassay testing in Côte d’Ivoire to investigate three questions concerning temporal trends in insecticide resistance. Although as noted in the Results, and discussed below, some important caveats must be considered, our analyses suggest that: the prevalence of resistance to each insecticide class has increased over time (question 1); but that this change is not uniform across insecticides and ecological zones (question 2); and, that whilst records of multiple resistance across insecticide classes do appear to have increased, this might be explained by an increase in the number of classes typically tested (question 3).

Striking differences in temporal trends among insecticides were observed between the two largest ecological zones, with a significantly stronger decline in bioassay mortality for DDT and each of the class I and II pyrethroids in ecological zone 1, but the reverse pattern for carbamates and organophosphates. This zonal effect might potentially be linked to differences in the use of insecticide in agriculture. Indeed, with the rank of first worldwide cocoa producer, third coffee producer and first rubber producer in Africa, and extensive rice cultivation across the country, agriculture remains the key component of the national economy. The distribution of agricultural areas was previously mapped in the country [13] and is particularly well developed in ecological zone 1 where insecticides are applied extensively [13]. However, data on which insecticide classes have been most commonly used in agriculture are unavailable, and so it is not possible to evaluate whether zonal shifts in the relative usage of different classes has occurred.

In Côte d’Ivoire an early investigation of the impact of pyrethroid resistance in *An. gambiae*, which compared mosquitoes from susceptible and resistant populations, suggested that ITN efficacy was not compromised [23]. Yet widespread use of insecticide-treated bednets has only occurred in the last five years [2] and it is possible that individual levels of resistance, rather than just prevalence (as recorded in all the studies we surveyed) may only have increased relatively recently. Assessments of contemporary insecticide resistance on LLINs and IRS are required to evaluate this. However, extensive use of insecticides in the past for other public health programmes may be a factor contributing to resistance trends. Successful control targeting onchocerciasis and trypanosomiasis vectors (*Glossina spp.*) from 1966 to 1983 eliminated onchocerciasis as a public health problem from the region crossed by the Bandaman River (from ecological zone 3 to 1). For this purpose, high amounts of DDT, pyrethroids, carbamates, and organophosphates were sprayed on a large geographical scale [24] and may have played a role in selecting for the resistance in malaria vectors currently seen today [25,26]. Unfortunately, no systematic monitoring programme based on insecticide accumulation in water and soil, (perhaps particularly pertinent for DDT) has been conducted and hence the longer-term selection pressure exerted by these activities is unknown.

One clear factor potentially linked to the difference in resistance trends is sharing of target sites, with pyrethroids and DDT both targeting the *para* voltage-gated sodium and carbamates and organophosphates targeting acetylcholinesterase. In general, *An gambiae* M (known as *An. Coluzzi*) and S (*An. gambiae s.s*) forms [27] were sympatric in all ecological zones but with varying relative frequencies. M form *An. gambiae* are particularly common in ecological zone 1, which showed the stronger declines in pyrethroid and especially DDT mortality; resistance to the latter being more strongly linked to *kdr* 1014F in M forms in Côte d’Ivoire [7]. It thus seems plausible that the dramatic rise in *kdr* 1014F frequency in M forms in Côte d’Ivoire, also documented in surrounding countries [28,29], could be at least a partial contributory factor. Our results highlight the critical importance of separating resistance testing results even between these closely related forms (now known as species). The apparent difference in trends between ecological zones for carbamates and organophosphates must be treated with considerable caution because it is evident that the decline in zone 2 is driven largely by high mortality in collections from the 1990s, which were not performed in zone 1 (see Figure 4).

Characterisation of spatial variance according to ecological zones is extremely coarse, with many areas under-represented or all-but unexplored, in addition to our analyses ignoring smaller scale spatial variation in resistance testing over time. Two reasons could explain the highly uneven distribution of bioassay data. The first reason could be the existence of experimental huts and the proximity to research institutes. During the first decade, the main experimental huts available in the country were located in ecological zone 2 in Bouake area. The huts were supervised by the Pierre Richet Institute, where several projects targeting malaria control have been conducted. These experimental huts are still in use and additional new huts in the Tiassalé area were built by the Swiss Centre of Scientific Research (Centre Suisse de Recherches Scientifiques, CSRS) in ecological zone 1 during the second decade. Overall in this and any other studies of temporal trends in insecticide resistance, sample-site specific biases are difficult to control and represent a major limiting factor.

Another extremely important source of bias when evaluating temporal trends in insecticide resistance is apparent from our analysis of multiple resistance. Typically maps representing insecticide resistance simply record presence or absence of resistance according to WHO criteria and do not explicitly consider spatial temporal variations in investigation effort. Yet this may explain much of the apparent pattern of increasing multiple resistance in Côte d’Ivoire; studies testing more classes are clearly more likely to detect multiple resistance. This does not in any way downplay the important consequences implied by detection of multiple resistance [7,30]. However, given that causation will inevitably be linked to correlation because of the limited temporal and spatial scale of properly controlled studies, it does highlight the importance of objective evaluation of available data.

## Conclusion

This is the first nationwide mapping study to attempt a synthesis of the 20 years of insecticide resistance data from Côte d’Ivoire, and we clearly show increasing prevalence of resistance albeit with a non-uniform pattern across areas and insecticides. Our study highlights the need for well-standardised, regular monitoring of resistance to multiple insecticide classes in Côte d’Ivoire, encompassing a wider spatial scale than is currently represented by the malaria-monitoring sentinel sites, as performed in other countries e.g. [31]. For several areas, susceptibility data on pyrethroid, organochlorine, organophosphate and carbamate need to be updated. For Tiassalé the high prevalence of resistance (as measured by the one hour bioassay exposure) does also signify high individual resistance levels, at least for deltamethrin and bendiocarb [7]. However, this cannot be assumed to be true generally [32], and requires evaluation to give data that may more closely align to the operationally-significant resistance of immediate concern for decision-makers. Since IRS is being suggested as a complementary tool to LLINs in the country, careful evaluation at local scale before implementation of IRS is needed to design an appropriate insecticide resistance management plan to combat spread of multi-class resistance known to be present at least locally in southern Côte d’Ivoire.

## Additional files

Additional file 1:
**Prevalence of insecticide resistance in** ***An. gambiae s.s*** **from Sikensi, Divo, Agboville and Tiassale during wet period in 2013 in Côte d’Ivoire.**
Additional file 2:
**Summaries of insecticide resistance data collected between 1993 and 2013 in Côte d’Ivoire (a), sampling zone and insecticide coverage (b) molecular form data (c) and** ***kdr*** **1014F data (d).**
Additional file 3:
**Distribution of pyrethroid resistance in Côte d’Ivoire between 1993–2002 (A) and 2003–2012 (B).**
Additional file 4:
**Distribution of DDT resistance in Côte d’Ivoire between 1993–2002 (A) and 2003–2012 (B).**
Additional file 5:
**Distribution of carbamate resistance in Côte d’Ivoire between 1993–2002 (A) and 2003–2012 (B).**
Additional file 6:
**Distribution of organophosphate resistance in Côte d’Ivoire between 1993–2002 (A) and 2003–2012 (B).**

## Abbreviations

ITNs: Insecticide treated nets
IRS: Indoor residual spraying
GIS: Geographical Information Systems (GIS)
IR: Insecticide resistance
DDT: Dichlorodiphenyltrichloroethane
*kdr* 1014F: Knockdown resistance mutation
M or S: Anopheles gambiae molecular forms identified as *An. coluzzii* and *An. gambiae s.s.*
LLINs: Lasting insecticidal nets
WHO: World Health Organization
GPIRM: Global Plan for Insecticide Resistance Management
ACT: Artemisinin combination therapy
GLiM: Generalized linear model (GLiM)
Ecozone: Ecological zones
Ace1^R^: Acetylcholinesterase
IPR: Pierre Richet Institute
CSRS: Centre Suisse de Recherches Scientifiques

## References

[1] WHO (2011). World Malaria Report 2011.

[2] WHO (2013). World Malaria Report 2013.

[3] WHO (2012). Global Plan for Insecticide Resistance Management in Malaria Vectors.

[4] Ranson H, N’Guessan R, Lines J, Moiroux N, Nkuni Z, Corbel V (2011). Pyrethroid resistance in African anopheline mosquitoes: what are the implications for malaria control?. Trends Parasitol.

[5] Aïzoun N, Aïkpon R, Gnanguenon V, Oussou O, Agossa F, Gil Germain Padonou GG, Akogbéto M (2013). Status of organophosphate and carbamates resistance in *Anopheles gambiae* sensu lato from the south and north Benin, West Africa. Parasit Vectors.

[6] Oduola AO, Idowu ET, Oyebola MK, Adeogun AO, Olojede JB, Otubanjo OA, Awolola TS (2012). Evidence of carbamate resistance in urban populations of Anopheles gambiae s.s. mosquitoes resistant to DDT and deltamethrin insecticides in Lagos, South-Western Nigeria. Parasit Vectors.

[7] Edi CV, Koudou GB, Jones CM, Weetman D, Ranson H (2012). Multiple-insecticide resistance in *Anopheles gambiae* mosquitoes, Southern Cote d’Ivoire. Emerg Infect Dis.

[8] Essandoh J, Yawson A, Weetman D (2013). Acetylcholinesterase (Ace-1) target site mutation 119S is strongly diagnostic of carbamate and organophosphate resistance in Anopheles gambiae s.s. and Anopheles coluzzii across southern Ghana. Malar J.

[9] Koffi AA, Ahoua Alou LP, Kabran J-PK, N’Guessan R, Pennetier C (2013). Re-visiting insecticide resistance status in *Anopheles gambiae* from Cote d’Ivoire: a nation-wide informative survey. PLoS One.

[10] Alou LPA, Koffi AA, Adja MA, Tia E, Kouassi PK, Kone M, Chandre F (2010). Distribution of ace-1(R) and resistance to carbamates and organophosphates in Anopheles gambiae s.s. populations from Côte d’Ivoire. Malar J.

[11] Bagayoko MAB, Faye O, Lymo E, Govere J, Gebremariam M, Manga L (2005). The status of malaria vector resistance to insecticides used for public health in the African region. WHO-AFRO: Commun Dis Bull Afr Region.

[12] Chandre F, Darriet F, Manguin S, Brengues C, Carnevale P, Guillet P (1999). Pyrethroid cross resistance spectrum among populations of *Anopheles gambiae s.s.* from Côte d’Ivoire. J Am Mosq Control Assoc.

[13] Chandre F, Darriet F, Manga L, Akogbeto M, Faye O, Mouchet J, Guillet P (1999). Status of pyrethroid resistance in *Anopheles gambiae sensu lato*. Bull World Health Organ.

[14] Koffi AA, Darriet F, N’Guessan R, Doannio JM, Carnevale P (1999). Laboratory evaluation of alpha-cypermethrin insecticide efficacy on *Anopheles gambiae* populations of Côte d’Ivoire resistant to permethrin and deltamethrin. Bull Soc Pathol Exot.

[15] Elissa N, Mouchet J, Riviere F, Meunier JY, Yao K (1993). Resistance of *Anopheles gambiae s.s* to pyrethroids in Côte d’Ivoire. Ann Soc belge Med Trop.

[16] N’Guessan R, Darriet F, Guillet P, Carnevale P, Traore-Lamizana M, Corbel V, Koffi AA, Chandre F (2003). Resistance to carbosulfan in *Anopheles gambiae* from Ivory Coast, based on reduced sensitivity of acetylcholinesterase. Med Vet Entomol.

[17] Koffi AA, Alou LP, Adja MA, Koné M, Chandre F, N’guessan R (2012). Update on resistance status of Anopheles gambiae s.s. to conventional insecticides at a previous WHOPES field site, “Yaokoffikro”, 6 years after the political crisis in Côte d’Ivoire. Parasit Vectors.

[18] Aregheore EM (2009). Côte d’Ivoire. Food and Agriculture Organization for the United Nation.

[19] Goula BT, Brou K, Brou T, Savannah I, Vamoryba F, Bernard S (2007). Estimation of daily extreme rainfall in the tropics: the case of Côte d’Ivoire by comparing Lognormal and Gumbel laws. Hydrolog Sci J.

[20] WHO (2013). Test Procedures for Insecticide Resistance Monitoring in Malaria Vectors.

[21] USGS 2008: United States Geological Survey (2008). Normalized Difference Vegetation Index: LandDAAC MODIS version_005 WAF NDVI.

[22] **Directory of Cities and Towns in the World, Global Gazetteer Version 2.1.** Alphabetical listing of Places in Côte d'Ivoire (Ivory Coast). http://www.fallingrain.com/world/IV/ Accessed 13 April 2014.

[23] Darriet F, N’Guessan R, Koffi AA, Konan L, Doannio JM, Chandre F, Carnevale P (2000). Impact of pyrethrin resistance on the efficacy of impregnated mosquito nets in the prevention of malaria: results of tests in experimental cases with deltamethrin SC. Bull Soc Pathol Exot.

[24] WHO (1985). Ten Years of Onchocerciasis Control: Review of the Work of the Onchocerciasis Control Programme in the Volta River Basin Area from 1974 to 1984. OCP/GVA/85. 1A.

[25] Le Berre R, Juge E, Rossolin P, Grébaut S (1967). FED - OCCGE Campaign for Onchocerciasis Vector Control: Target Site of Sikasso (Mali), Tiassalé et Korhogo (Côte d’Ivoire). Technical Conference OCCGE.

[26] Koeman JH, Balk F, Takken W: **The Impact of Tsetse Control Operations. A Report on Present Knowledge. FAO Paper, 7 Rev1.** Rome: United Nations Organization for Food and Agriculture; 1980.

[27] Coetzee M, Hunt RH, Wilkerson R, Della Torre A, Coulibaly MB, Besansky NJ (2013). Anopheles coluzzii and Anopheles amharicus, new members of the Anopheles gambiae complex. Zootaxa.

[28] Dabire KR, Diabate A, Agostinho F, Alves F, Manga L, Faye O, Baldet T (2008). Distribution of the members of *Anopheles gambiae* and pyrethroid knock-down resistance gene (*kdr*) in Guinea-Bissau, West Africa. Bull Soc Pathol Exot.

[29] Lynd A, Weetman D, Barbosa S, Egyir Yawson A, Mitchell S, Pinto J, Hastings I, Donnelly MJ (2010). Field, genetic, and modeling approaches show strong positive selection acting upon an insecticide resistance mutation in *Anopheles gambiae* s.s. Mol Biol Evol.

[30] Edi CV, Djogbenou L, Jenkins AM, Regna K, Muskavitch MAT, Poupardin R, Jones CM, Essandoh J, Ketoh GK, Paine MJI, Koudou GB, Donnelly MJ, Ranson H, Weetman D (2014). *CYP6* P450 enzymes and *ACE-1* duplication produce extreme and multiple insecticide resistance in the malaria mosquito anopheles gambiae. PLoS Genet.

[31] Kabula BI, Attah PK, Wilson MD, Boakye DA (2011). Characterization of *Anopheles gambiae s.l.* and insecticide resistance profile relative to physicochemical properties of breeding habitats within Accra Metropolis, Ghana. Tanzan J Health Res.

[32] Toé KH, Jones CM, N’Fale S, Ismail HM, Dabiré RK, Ranson H (2014). Increased pyrethroid resistance in malaria vectors and decreased bednet efficacy in Burkina Faso. Emerg Infect Dis.

