# Supplementary material for: Long-term trends in Anopheles gambiae insecticide resistance in Côte d’Ivoire
Source: Parasit Vectors. 2014 Nov 28;7:500. doi: 10.1186/s13071-014-0500-z (PMC4269959; doi:10.1186/s13071-014-0500-z)
Supplement: Additional file 1: — Prevalence of insecticide resistance in An. gambiae s.s from Sikensi, Divo, Agboville and Tiassale during wet period in 2013 in Côte d’Ivoire. [file 13071_2014_500_MOESM1_ESM.pdf]

Additional file 1. Prevalence of insecticide resistance in An. gambiae s.s from Tiassalé, Sikensi, Divo and Agboville in 2013 in Côte d'Ivoire.

Strains	Insecticides	No. tested	Dead	Mortality (%)	status
Sikensi	bendiocarb (0.1%)	108	61	56.5 (46.6-66.0)	R
	fenitrothion (1.0%)	111	99	89.2 (81.9-94.3)	R
	Deltamethrin (0.05%)	81	42	51.8 (40.5-63.1)	R
Divo	bendiocarb (0.1%)	199	155	77.9 (71.5-83.4)	R
	fenitrothion (1.0%)	196	192	98.0 (94.9-99.4)	S
	Deltamethrin (0.05%)	99	64	64.6 (54.4-74.0)	R
	primiphos methyl (0.25%)	48	21	43.8 (29.5-58.8)	R
Agboville	bendiocarb (0.1%)	116	8	6.9 (3.0-13.1)	R
	fenitrothion (1.0%)	103	64	62.1 (52.0-71.5)	R
	Deltamethrin (0.05%)	116	73	62.9 (53.5-71.7)	R
	primiphos methyl (0.25%)	94	15	16.0 (9.2-24.9)	R
Tiassale	primiphos methyl (0.2%)	100	63	63.0 (52.8-72.4)	R
	primiphos methyl (0.25%)	100	68	68.0 (57.9-77)	R

Data were collected by the AvecNet research programme between May 2012 and March 2013. All percentages are calculated from total mosquito tested (No. tested) with 95% confidence intervals. Resistant population = R; suspected resistant population = R/S
