# Supplementary figures and images for: Long-term trends in Anopheles gambiae insecticide resistance in Côte d’Ivoire
Source: Parasit Vectors. 2014 Nov 28;7:500. doi: 10.1186/s13071-014-0500-z (PMC4269959; doi:10.1186/s13071-014-0500-z)

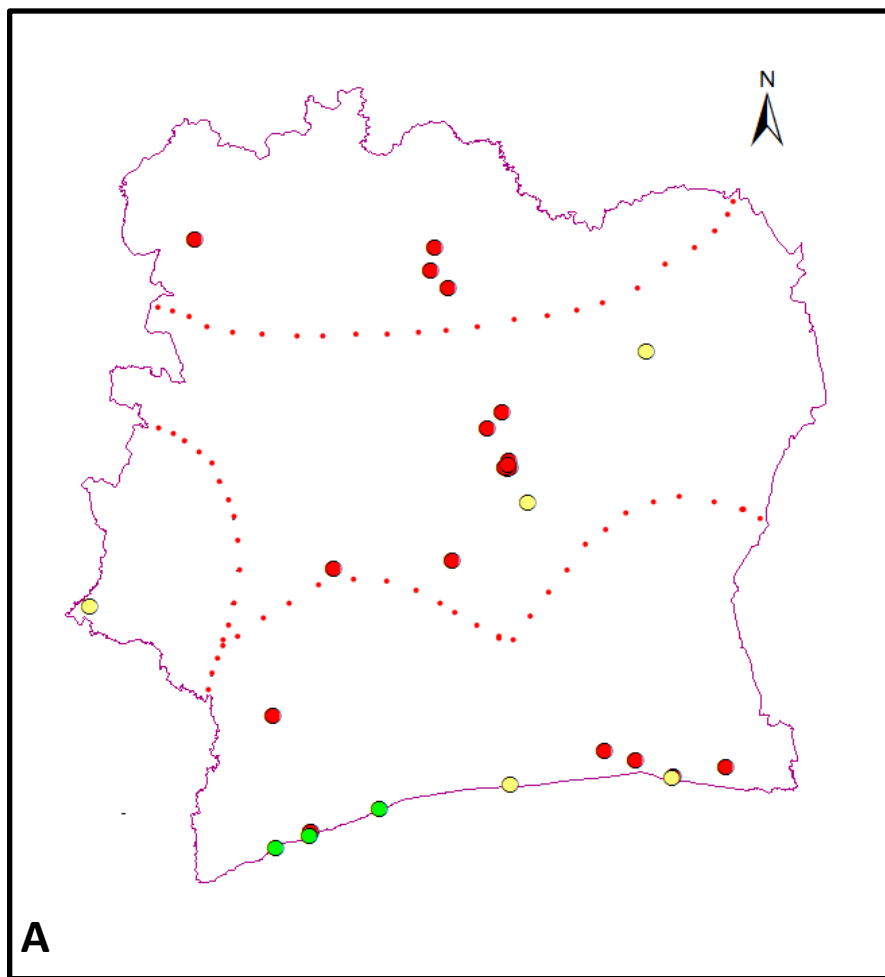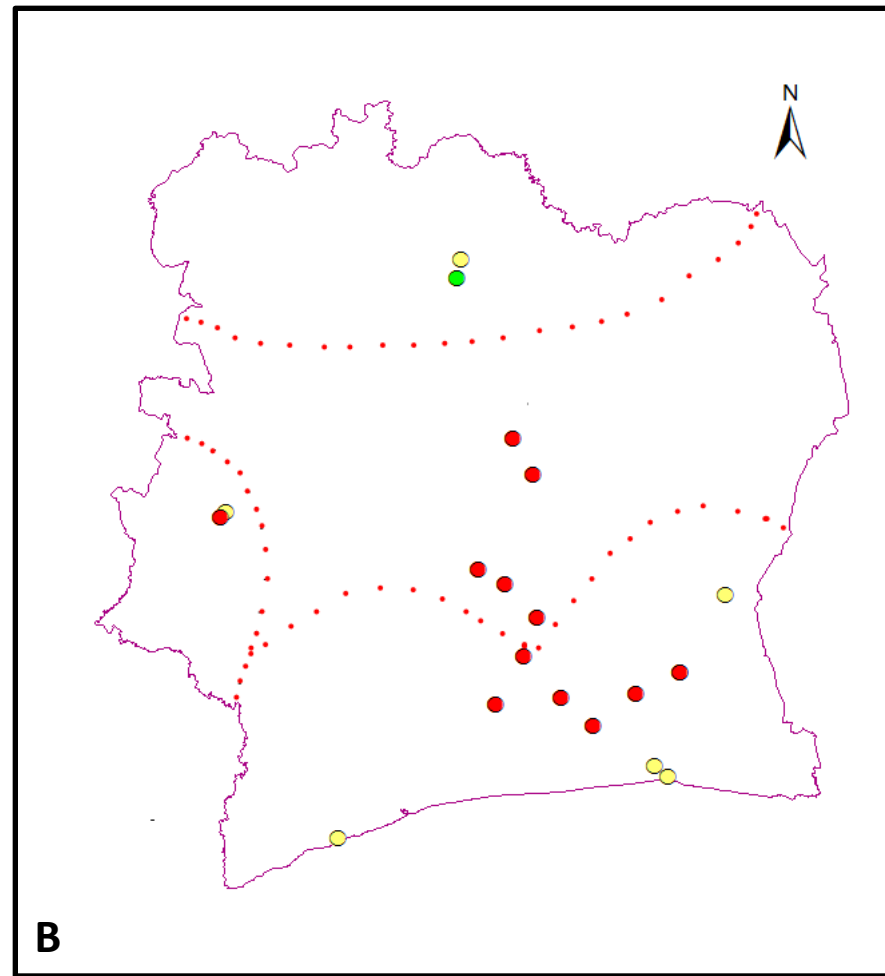

- 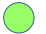 No resistance
- 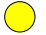 Suspected resistance
- 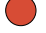 Confirmed resistance

0 55 110 220 Kilometers

Supplement: Additional file 3: — Distribution of pyrethroid resistance in Côte d’Ivoire between 1993–2002 (A) and 2003–2012 (B). [file 13071_2014_500_MOESM3_ESM.pdf]

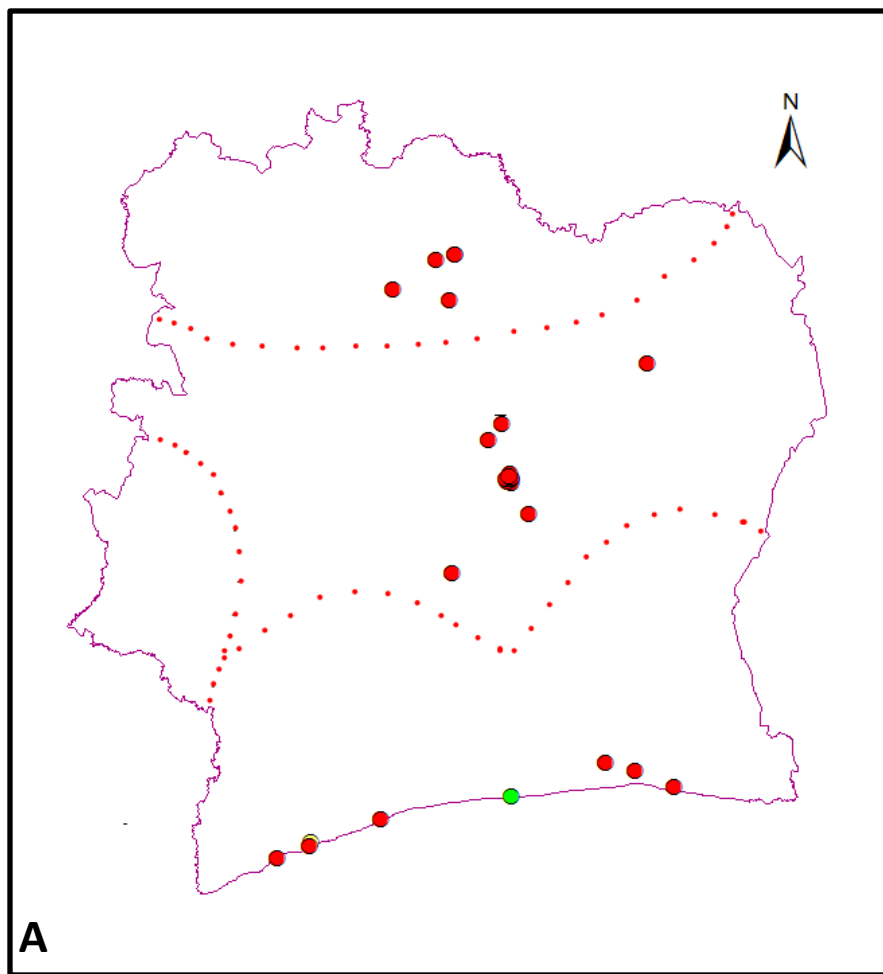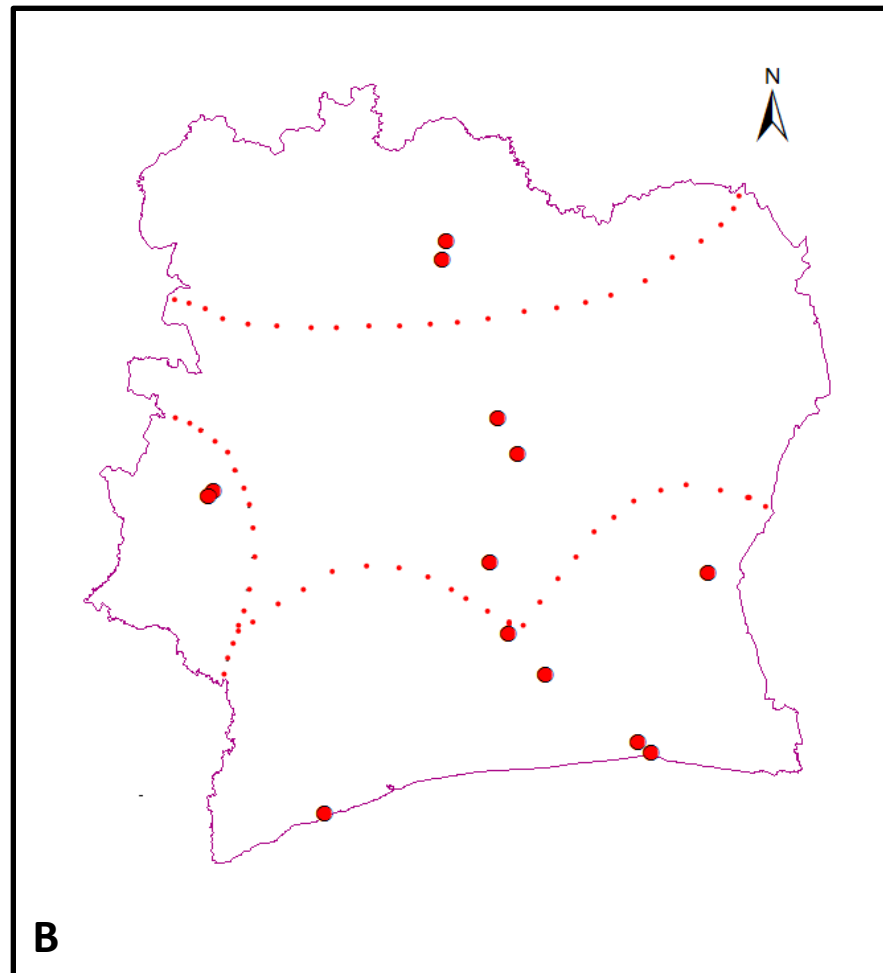

0 55 110 220 Kilometers

- No resistance
- Suspected resistance
- Confirmed resistance

Supplement: Additional file 4: — Distribution of DDT resistance in Côte d’Ivoire between 1993–2002 (A) and 2003–2012 (B). [file 13071_2014_500_MOESM4_ESM.pdf]

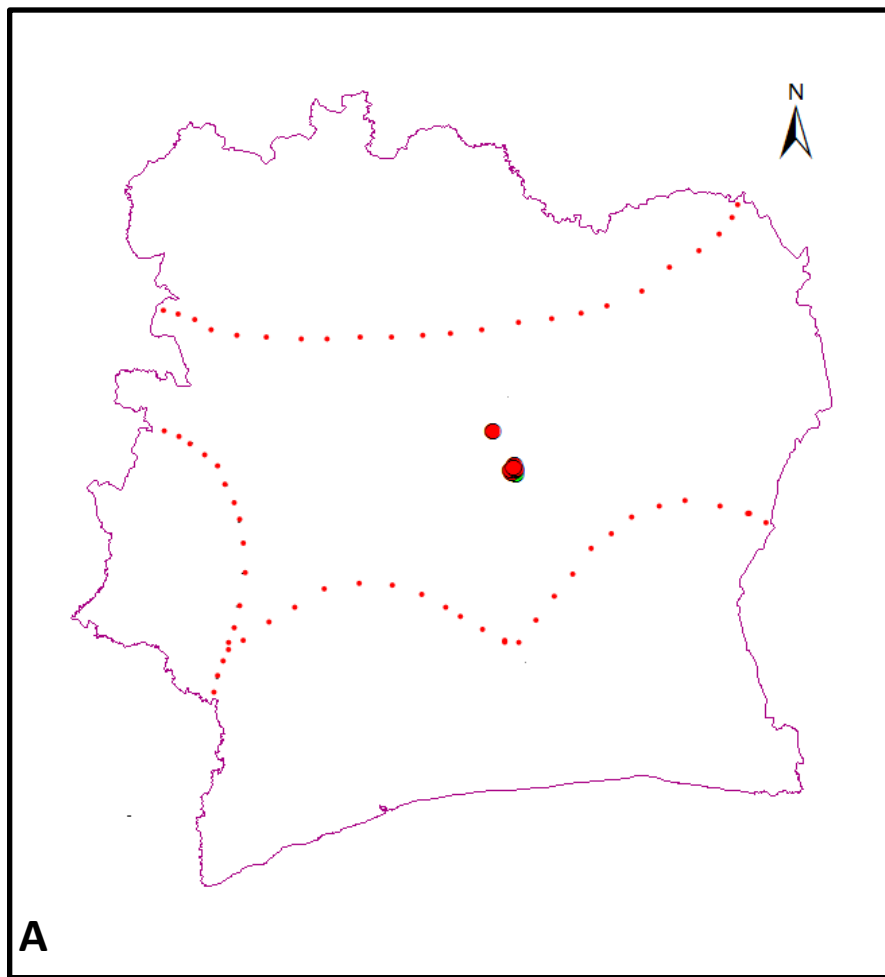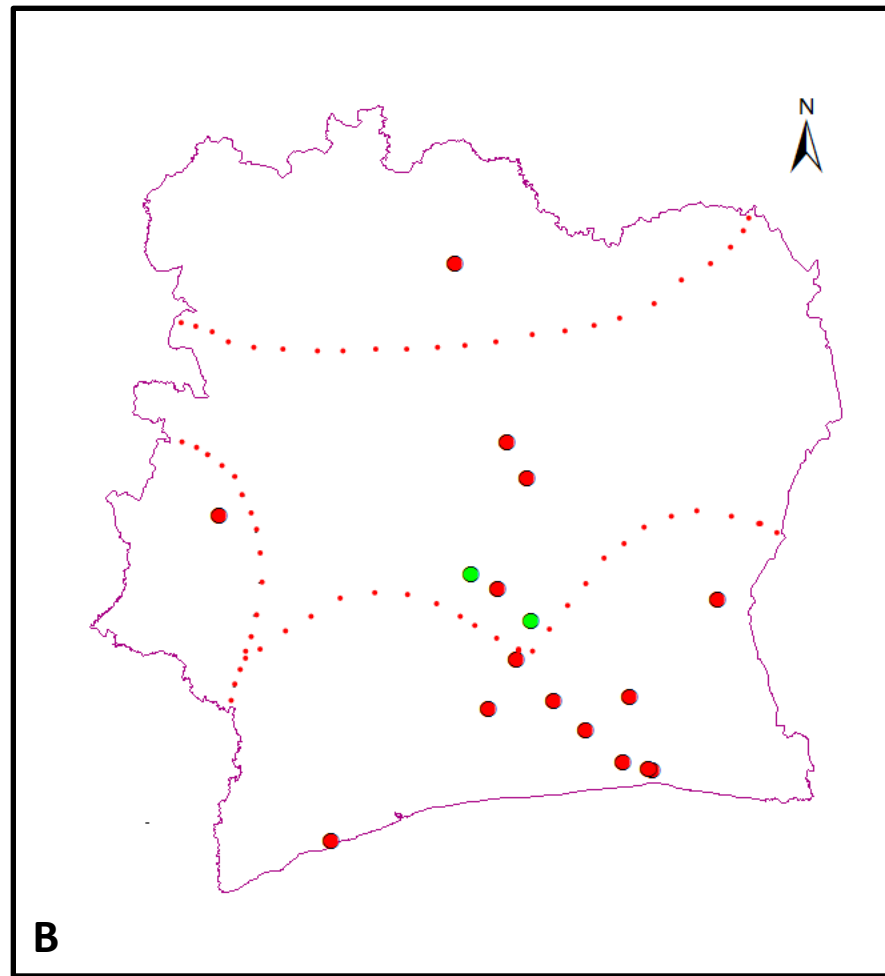

0 55 110 220 Kilometers

- No resistance
- Suspected resistance
- Confirmed resistance

Supplement: Additional file 5: — Distribution of carbamate resistance in Côte d’Ivoire between 1993–2002 (A) and 2003–2012 (B). [file 13071_2014_500_MOESM5_ESM.pdf]

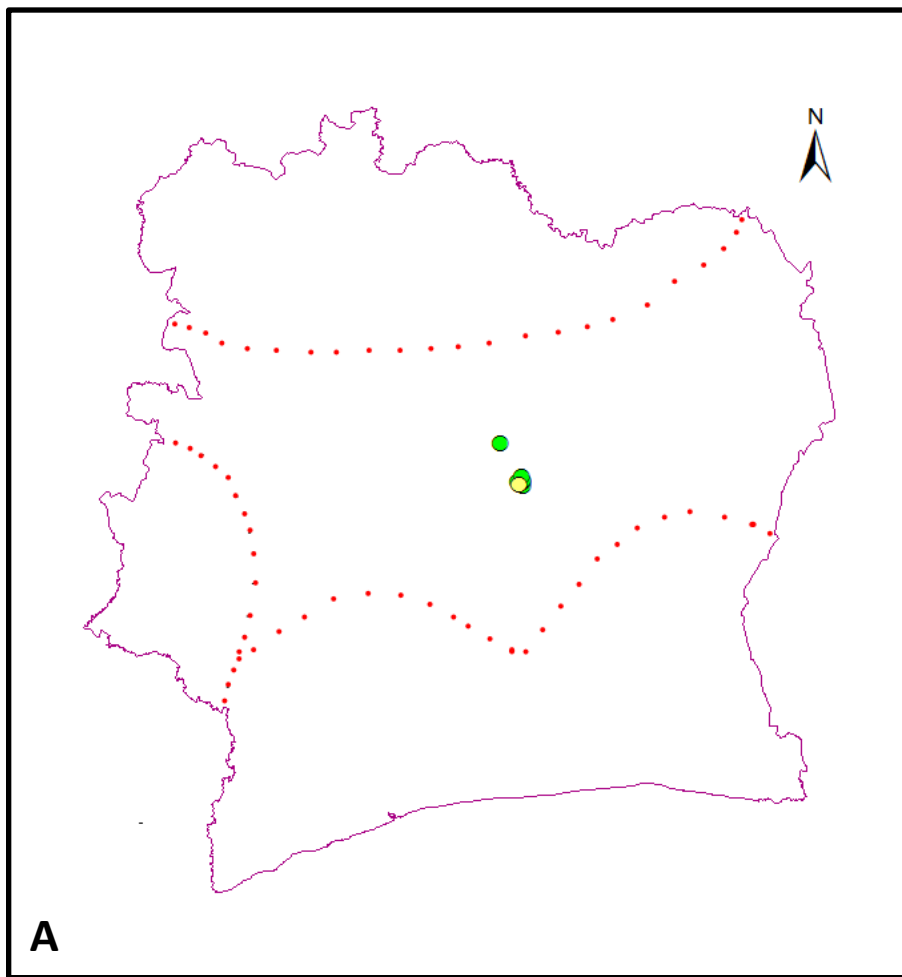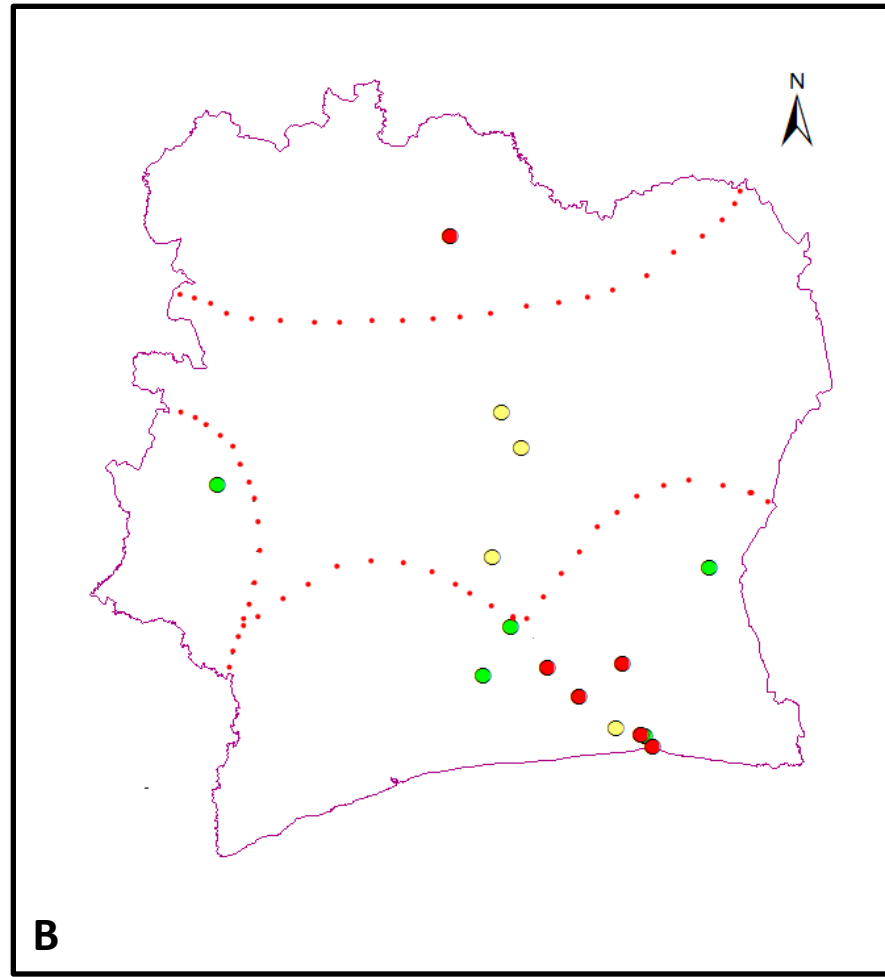

0 55 110 220 Kilometers

- No resistance
- Suspected resistance
- Confirmed resistance

Supplement: Additional file 6: — Distribution of organophosphate resistance in Côte d’Ivoire between 1993–2002 (A) and 2003–2012 (B). [file 13071_2014_500_MOESM6_ESM.pdf]
